# Clinical study on the diagnosis of porcine streptococcal meningitis with negative blood and cerebrospinal fluid culture by next-generation sequencing

**DOI:** 10.1186/s40001-021-00554-2

**Published:** 2021-08-03

**Authors:** Eryi Zhao, Daimei Wang, Na Li, Shixiong Huang, Zhongyan Zhao, Shijun Hu, Xiangying He, Guoqiang Wen

**Affiliations:** 1 Department of Neurology, Hainan General Hospital, Hainan Affilicated Hospital of Hainan Medical University, Haikou, 570311 Hainan China; 2 Department of Pharmacy, Hainan General Hospital, Hainan Affilicated Hospital of Hainan Medical University, Haikou, 570311 Hainan China; 3 Department of Pharmacy, Qianjiang Central Hospital of Chongqing, No. 63 of Road 9, Chengxi Street, Qianjiang District, Chongqing, 409099 China

**Keywords:** Diagnosis, Next-generation sequencing, Streptococcal meningitis

## Abstract

**Background:**

Streptococcus suis (Ss) is a Gram-positive and anaerobic zoonotic pathogen that is susceptible to all populations and can cause meningitis, septicemia, endocarditis and arthritis in humans.

**Methods:**

In this study, patients with meningitis who were admitted to our hospital with negative blood and cerebrospinal fluid culture were divided into a next-generation sequencing group and a control group. In the next-generation sequencing group, we used the next-generation sequencing method to detect pathogenic bacteria in the patients’ cerebrospinal fluid. In the control group, we used blood and cerebrospinal fluid bacterial culture method to detect pathogenic bacteria in the patients' cerebrospinal fluid. The detection rates of pathogenic bacteria in the cerebrospinal fluid of the two groups were compared and analyzed.

**Results:**

A total of 18 patients were included in this study, including 8 patients in the next-generation sequencing group and 10 patients in the control group. The mean age (*P* = 0.613) and mean disease duration (*P* = 0.294) were similar in both groups. Patients in the next-generation sequencing group had a leukocyte count of 13.13 ± 4.79 × 10^9^, a neutrophil percentage of 83.39 ± 10.36%, and a C-reactive protein level of 134.95 ± 107.69 mg/L. Patients in the control group had a temperature of 38.32 ± 1.07, a leukocyte count of 8.00 ± 2.99 × 10^9^, and a neutrophil percentage of 74.61 ± 8.89%, and C-reactive protein level was 4.75 ± 6.8 mg/L. The statistical results showed that the leukocytes (*P* = 0.013) and C-reactive protein levels (*P* = 0.001) were significantly higher in the patients of the next-generation sequencing group than in the control group. No statistically significant differences were seen in body temperature and neutrophil percentage between the two groups (*P* > 0.05). The incidence of intracranial pressure and meningeal irritation signs were similar in the two groups (*P* > 0.05). The detection rate of Streptococcus suis in the cerebrospinal fluid of patients in the next-generation sequencing group was 100%, and the detection rate of Streptococcus suis in the cerebrospinal fluid of the control group was 0%.

**Conclusion:**

The detection rate of Streptococcus suis infection in cerebrospinal fluid by next-generation sequencing was significantly higher than that by blood and cerebrospinal fluid bacterial culture. Therefore, the diagnosis of porcine streptococcal meningitis by next-generation sequencing method is worthy of clinical promotion and application.

## Introduction

Streptococcus suis (Ss) is a Gram-positive and anaerobic zoonotic pathogen that is susceptible to all populations and can cause meningitis, septicemia, endocarditis and arthritis in humans. Among them, meningitis is the most common, and most of them can have severe sequelae of cochlear and vestibular nerve damage [1]. Its invasion of the central nervous system through the blood–brain barrier or blood-cerebrospinal fluid barrier via cerebral microvascular epithelial cells or choroidal epithelial cells leads to inflammation of the meningeal brain parenchyma [2], and the main causative risk factors include occupational exposure to pigs, raw pork, or consumption of raw pig meat products [3].

Cerebrospinal fluid pathogenic bacteria with next-generation sequencing (NGS) technology is an emerging molecular diagnostic method for rapid detection of intracranial pathogens [4], which has unique advantages in unexplained pathogenic infections. Therefore, this study intends to investigate the diagnostic value of next-generation sequencing (NGS) in the diagnosis of porcine streptococcal meningitis with negative blood and cerebrospinal fluid culture.

## Data and methods

### Study subjects

Patients with meningitis who were admitted to our hospital with negative blood and cerebrospinal fluid culture were the main subjects of this study, and were divided into next-generation sequencing group and control group. This study is in accordance with the *Declaration of Helsinki of the World Medical Association* and has been approved by the ethics committee of our hospital, and all patients signed an informed consent form.

### Inclusion and exclusion criteria

Inclusion criteria are: (1) patients diagnosed with meningitis; (2) age > 18 years; and (3) patients who had signed the informed consent form. Exclusion criteria are: (1) patients with advanced malignancy and (2) patients with incomplete information.

### Study methods

In the next-generation sequencing group, we used the next-generation sequencing method to detect pathogenic bacteria in the patients’ cerebrospinal fluid by next-generation sequencing equip (USA, Illumina Nextseq550). In the control group, we used blood and cerebrospinal fluid bacterial culture method to detect pathogenic bacteria in the patients’ cerebrospinal fluid. The detection rates of pathogenic bacteria in the cerebrospinal fluid of patients in the two groups were compared and analyzed.

### Cerebrospinal fluid detection method

About 2–3 mL of cerebrospinal fluid was collected by lumbar puncture and stored at − 20 °C after aseptic sealing. DNA was extracted according to the DNA extraction kit (Tiangen (Beijing) Biotech Co., Ltd, Tiangen DNA Mini kit DP316). Next-generation sequencing (NGS) technology was used to detect pathogens in the cerebrospinal fluid.

### Main observation indexes

The main observation indexes of this study included gender, age, disease duration, body temperature, leukocytes, neutrophil percentage, C-reactive protein level, intracranial pressure, and cerebrospinal fluid test.

### Statistical analysis

SPSS 20.0 statistical software was used for data processing in this study, and the measurement data were expressed as mean ± standard deviation (${\overline{\text{x}}}\, \pm \,{\text{s}}$). Count data were expressed as percentages (%). The *t* test was used for comparison between two groups obeying normal distribution; the non-parametric test was used for comparison between groups not obeying normal distribution. Counting data were tested by Chi-square test. *P* < 0.05 was considered a statistically significant difference.

## Results

### General information

A total of 18 patients were included in this study, including 8 patients in the next-generation sequencing group and 10 patients in the control group. The mean age of patients in the next-generation sequencing group was 54.25 ± 8.60 years, and the mean duration of disease was 5.88 ± 4.64 days. The mean age of patients in the control group was 50.10 ± 21.31 years, and the mean disease duration was 9.50 ± 8.46 days. There was no significantly difference about mean age (*P* = 0.613) and mean duration of illness (*P* = 0.294) between the two groups.

### Comparison of infection indicators between the two groups of patients

**Fig. 1:**
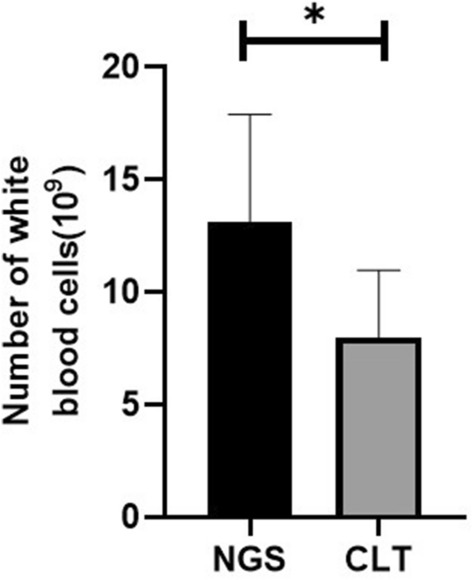
Comparison of leukocyte count between the two groups of patients

**Fig. 2:**
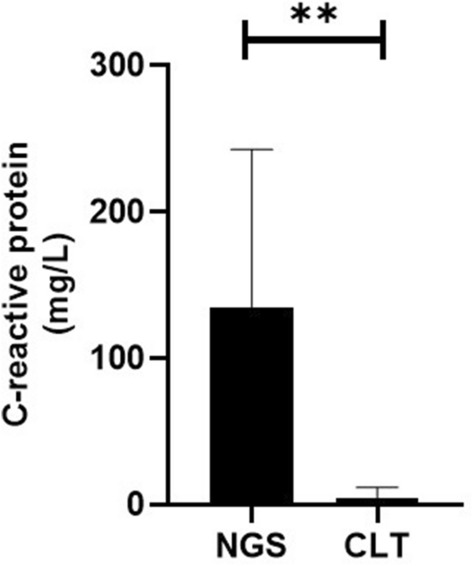
Comparison of C-reactive protein levels between the two groups of patients

The results showed that the white blood cell count was significantly higher in the diphtheria group than control group (13.13 ± 4.79 × 10^9^ vs. 8.00 ± 2.99 × 10^9^, *P* = 0.013), the C-reactive protein level was significantly higher in the diphtheria group than control group (134.95 ± 107.69 mg/L vs. 4.75 ± 6.8 mg/L, *P* = 0.001). The body temperature (38.95 ± 0.49 °C vs. 38.32 ± 1.07 °C, *P* = 0.143) and neutrophil percentage (83.39 ± 10.36% vs. 74.61 ± 8.89%, *P* = 0.071) were similar in both the groups (Figs. 1, 2).

### Results of cerebrospinal fluid testing in the two groups of patients

**Fig. 3:**
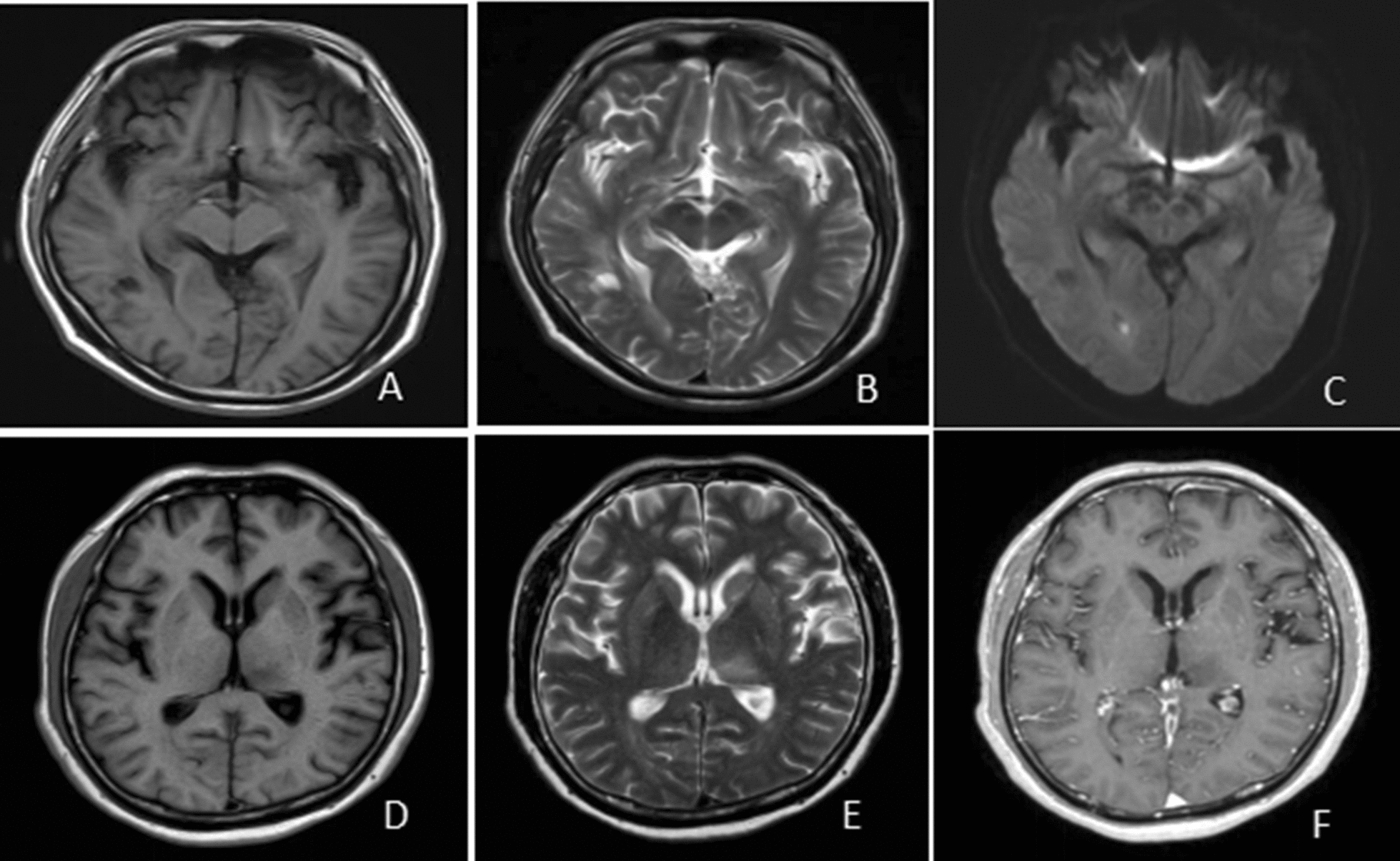
The results of cranial imaging of porcine streptococcal meningitis. **A**–**C** The cranial MRI results of one patient, showing an abnormal lesion in the right occipital lobe parietal ventricle with small abscess formation; **D**–**F** The cranial MRI results of another patient, showing inflammatory lesions in the left thalamus and basal ganglia region

The intracranial pressure of patients in the next-generation sequencing group was 205.63 ± 66.09 mmH2O, 190.70 ± 53.55 mmH2O in the control group. 100% of patients in the next-generation sequencing group and 80% of patients in the control group had meningeal irritation signs. The statistical results showed that the incidence of intracranial pressure and meningeal irritation signs were similar in the two groups (*P* > 0.05). The detection rate of Streptococcus suis in the cerebrospinal fluid of patients in the next-generation sequencing group was 100%, and the detection rate of Streptococcus suis in the cerebrospinal fluid of the control group was 0% (Fig. 3).

## Discussion

Porcine streptococcal meningitis is the most predominant type of human infection with porcine streptococcal disease, which has been outbreaks and epidemics in Sichuan and Jiangsu in China, but is disseminated in other provinces, and rare cases are reported in Hainan [5]. The diagnosis of this disease is based on the epidemiological history, clinical manifestations, and confirmation of diagnosis requires positive blood and cerebrospinal fluid culture of Streptococcus suis [6], but due to factors such as low blood and cerebrospinal fluid bacteria, insufficient sampling or pre-treatment with antibiotics, resulting in positive blood and cerebrospinal fluid culture is not high, the positive rate of blood bacterial culture is about 32%, the positive rate of cerebrospinal fluid bacterial culture is 18.5%, and negative blood and cerebrospinal fluid bacterial culture increases the difficulty of clinical diagnosis [7].

Common signs and symptoms of porcine streptococcal meningitis include fever, headache, and neck stiffness [8–11]. Bilateral hearing impairment occurs early in the disease and the incidence can be as high as 66.4% [12], mainly due to labyrinthitis. The cellular classification of the cerebrospinal fluid in this disease is dominated by multiple nucleated cells, which may be misdiagnosed as viral meningitis or tuberculous meningitis once it is quickly converted to single nucleated cells or lymphocytes predominant after antibiotic treatment [13].

Next-generation sequencing technology, as an emerging molecular diagnostic method in current clinical practice [14–18], has been applied in various infectious disease areas in clinical practice [19, 20]. Therefore, the present study was proposed to investigate the diagnostic value of next-generation sequencing (NGS) in blood and cerebrospinal fluid culture-negative porcine streptococcal meningitis. A total of 18 patients were included in this study, including 8 patients in the next-generation sequencing group and 10 patients in the control group. The results of the study showed that the detection rate of Streptococcus suis in the cerebrospinal fluid of patients in the next-generation sequencing group was 100%, and the detection rate of Streptococcus suis in the cerebrospinal fluid of the control group was 0%. Therefore, when the pathogenic bacteria cannot be detected by blood and cerebrospinal fluid bacterial culture methods, the pathogenic bacteria in cerebrospinal fluid can be detected by next-generation sequencing method. In this study, the results showed that the leukocytes and C-reactive protein levels were significantly higher in the patients of the next-generation sequencing group than control group. The reason maybe due to the specific clinical characteristics, such as complicated with other bacterial infections. However, other reasons still needs further research.

The present study still has the following shortcomings. First, this study is a single-center clinical study, and follow-up multicenter clinical studies are still needed for further investigation. Second, the sample size included in this study was small, and further studies with larger sample size are needed.

## Conclusion

The detection rate of Streptococcus suis infection in cerebrospinal fluid by next-generation sequencing was significantly higher than that by blood and cerebrospinal fluid bacterial culture. Therefore, when clinically suspected streptococcal meningitis, but the cerebrospinal fluid culture is negative, we can use this next generation of sequencing to confirm the diagnosis, so as to improve the clinical diagnostic efficiency of streptococcal meningitis. The diagnosis of Streptococcus suis meningitis by next-generation sequencing method is worthy of clinical application.

## Data Availability

All data generated or analyzed during this study are included in this published article.

## Abbreviations

NGS: Next-generation sequencing
Ss: Streptococcus suis
